# Barrier Island Morphology and Sediment Characteristics Affect the Recovery of Dune Building Grasses following Storm-Induced Overwash

**DOI:** 10.1371/journal.pone.0104747

**Published:** 2014-08-22

**Authors:** Steven T. Brantley, Spencer N. Bissett, Donald R. Young, Catherine W. V. Wolner, Laura J. Moore

**Affiliations:** 1 Department of Forest Resources, University of Minnesota, St. Paul, Minnesota, United States of America; 2 Department of Biology, Virginia Commonwealth University, Richmond, Virginia, United States of America; 3 Department of Environmental Sciences, University of Virginia, Charlottesville, Virginia, United States of America; 4 Department of Geological Sciences, University of North Carolina, Chapel Hill, North Carolina, United States of America; Dauphin Island Sea Lab, United States of America

## Abstract

Barrier islands are complex and dynamic systems that provide critical ecosystem services to coastal populations. Stability of these systems is threatened by rising sea level and the potential for coastal storms to increase in frequency and intensity. Recovery of dune-building grasses following storms is an important process that promotes topographic heterogeneity and long-term stability of barrier islands, yet factors that drive dune recovery are poorly understood. We examined vegetation recovery in overwash zones on two geomorphically distinct (undisturbed vs. frequently overwashed) barrier islands on the Virginia coast, USA. We hypothesized that vegetation recovery in overwash zones would be driven primarily by environmental characteristics, especially elevation and beach width. We sampled species composition and environmental characteristics along a continuum of disturbance from active overwash zones to relict overwash zones and in adjacent undisturbed environments. We compared species assemblages along the disturbance chronosequence and between islands and we analyzed species composition data and environmental measurements with Canonical Correspondence Analysis to link community composition with environmental characteristics. Recovering and geomorphically stable dunes were dominated by *Ammophila breviligulata* Fernaud (Poaceae) on both islands while active overwash zones were dominated by *Spartina patens* (Aiton) Muhl. (Poaceae) on the frequently disturbed island and bare sand on the less disturbed island. Species composition was associated with environmental characteristics only on the frequently disturbed island (*p* = 0.005) where *A. breviligulata* was associated with higher elevation and greater beach width. *Spartina patens*, the second most abundant species, was associated with larger sediment grain size and greater sediment size distribution. On the less frequently disturbed island, time since disturbance was the only factor that affected community composition. Thus, factors driving the abundance of dune-building grasses and subsequent recovery of dunes varied between the two geomorphically distinct islands.

## Introduction

Barrier islands host critically important ecosystems that provide a number of ecosystem services to coastal communities. The stability and function of these systems are threatened by sea-level rise [1] and the potential for coastal storms to increase in frequency and intensity [2], [3]. Barrier islands are complex and dynamic environments where steep environmental gradients (i.e. high spatial variability in resources and/or stresses), frequent disturbances and feedbacks between vegetation and topography interact to affect both community structure and geomorphic stability (i.e., the degree to which dune or overwash topography maintains form throughout time) [4], [5], [6]. Storm overwash events, where wave runup and storm surge combine to overtop dunes, are particularly important in driving both community composition and island topography [3], [6], [7], [8], [9], [10]. Overwash may reduce or temporarily eliminate vegetative cover by exposing plants in low-elevation areas (relative to wave height) and/or areas unprotected by dunes to severe, acute stresses such as saltwater flooding, abrasion by water-borne sand and sand burial [4], [11], [12], [13]. During severe storms, overwash may alter local topography by flattening dunes, a primary source of protection for many barrier island plants [6], [7], [8], [9], [14], [15].

Because of the interactions between vegetation and topography during disturbance and recovery, feedbacks between vegetation and abiotic processes, like dune-building, are an important part of ecosystem function in coastal systems [4], [16]. Recovery of local topography after severe overwash events depends on both the presence of dune-building grasses that trap sand and rebuild elevation and an adequate supply of sand for dune building [6], [16], [17]. On larger islands with diverse topographies, only severe storms can overtop and/or flatten dunes. Given sufficient time between storms, a protective dune (or dunes) may form if dune-building species are present in adequate densities and sediment supply is sufficient. On low-elevation, frequently disturbed islands the frequency of overwash and/or the absence of dune building grasses results in a lack of stable dunes, which further perpetuates a low elevation state [4], [5], [10]. Many of these processes are well documented; however, the specific mechanisms that drive recovery and composition of plant communities, or result in suppression of dune-building species, after overwash events have not been adequately described.

Along the Mid-Atlantic and northeastern coast of the United States, recovery of dunes after overwash depends largely on the establishment of the dune-building grass *Ammophila breviligulata* Fernaud (Poaceae). Commonly known as American beachgrass, *A. breviligulata* is characterized by a guerilla root morphology and high tolerance to sand burial that initiates the formation of a continuous line of protective dunes along coastlines [18], [19]. Colonization of overwash zones with *A. breviligulata* is a transitory, but necessary, step in ecosystem recovery after overwash events [16], [17]. Barrier island plants, especially species that colonize the seaward side of islands, are exposed to a number of harsh physical stresses such as sea spray, intense solar radiation and blowing sand, which limit the number of species that can inhabit this environment [11], [13], [20], [21], [22], [23], [24]. *Ammophila breviligulata* is one of a very few species that can thrive in this environment and are considered to be foundation species for these systems [25]; however, a lack of dune-building grasses in some low-lying areas suggests that other mechanisms, especially physical drivers, are associated with *A. breviligulata* abundance [10]. Other species can also tolerate the harsh physical conditions that characterize active overwash zones and presence of these species may have a negative effect on dune-building. Some of these species have been termed “burial-tolerant stabilizers”, because they can withstand sand burial like *A. breviligulata* and they tend to stabilize surface sediments in active overwash zones, but they do not trap sand to build dunes [5].

Coastal plant communities have often served as model systems to study the relative importance of physical, geographic, and biotic drivers of plant community assemblages. The relatively simple structure and clear delineations among vegetation types make coastal plant communities an ideal system to test hypotheses related to community assemblages. As a result of steep environmental gradients and visible delineations in vegetation, a niche model has long provided the best explanation of spatial patterns of species abundance in coastal systems [11], [20], [26]. More recently, a landscape position model was described that condenses a variety of key coastal environmental gradients into two variables—elevation above mean sea level and distance to shoreline— to create “habitat polygons” [24]. Habitat polygons serve as surrogates for a wide range of environmental variables and successfully described the distribution of dominant species (i.e. species highest in frequency and abundance) across a range of functional groups on a Virginia barrier island [24]. However, it is uncertain whether the habitat polygon for a given species represents a universal threshold of establishment or if habitat polygons vary among islands. Furthermore, there may be situations in which habitat polygons result from ecogeomorphic feedbacks that alter the landscape, such as dune-building after establishment of dune-building grasses. For example, *A. breviligulata*, which frequently inhabits low-lying, disturbed areas, actually increases elevation by trapping sand that accretes vertically to form dunes [17], [24]. Such feedbacks complicate inferences of cause and effect between habitat characteristics and community composition.

Our objectives were to examine variations in plant community structure across a disturbance chronosequence of overwash recovery on two geomorphically distinct barrier islands to determine how vegetation recovery varies with landscape-level island geomorphologic characteristics and local environmental characteristics. To support these objectives, we addressed three questions relating to community development in overwash zones: 1) How do plant communities vary among active, recovering and relict overwash zones and adjacent, undisturbed communities? 2) How does plant community recovery following overwash vary between two geomorphically distinct barrier islands? and 3) How does community composition relate to elevation, distance to shoreline, beach width and sediment characteristics? While we addressed these questions for the whole community, we give additional attention to two common dune species—*A. breviligulata* and *Spartina patens* (Aiton) Muhl. (Poaceae)—that have previously been shown to have important effects on dune building. We hypothesized that in overwash zones vegetation recovery, including recovery of *A. breviligulata*, would follow a predictable trajectory on both islands depending on environmental characteristics, especially elevation and distance to shoreline. These results contribute to our understanding of the relative importance of time since overwash, local environmental parameters and island geomorphology in the recovery of plant communities and recovery of dunes in storm overwash zones.

## Methods

### Study site

**Figure 1:**
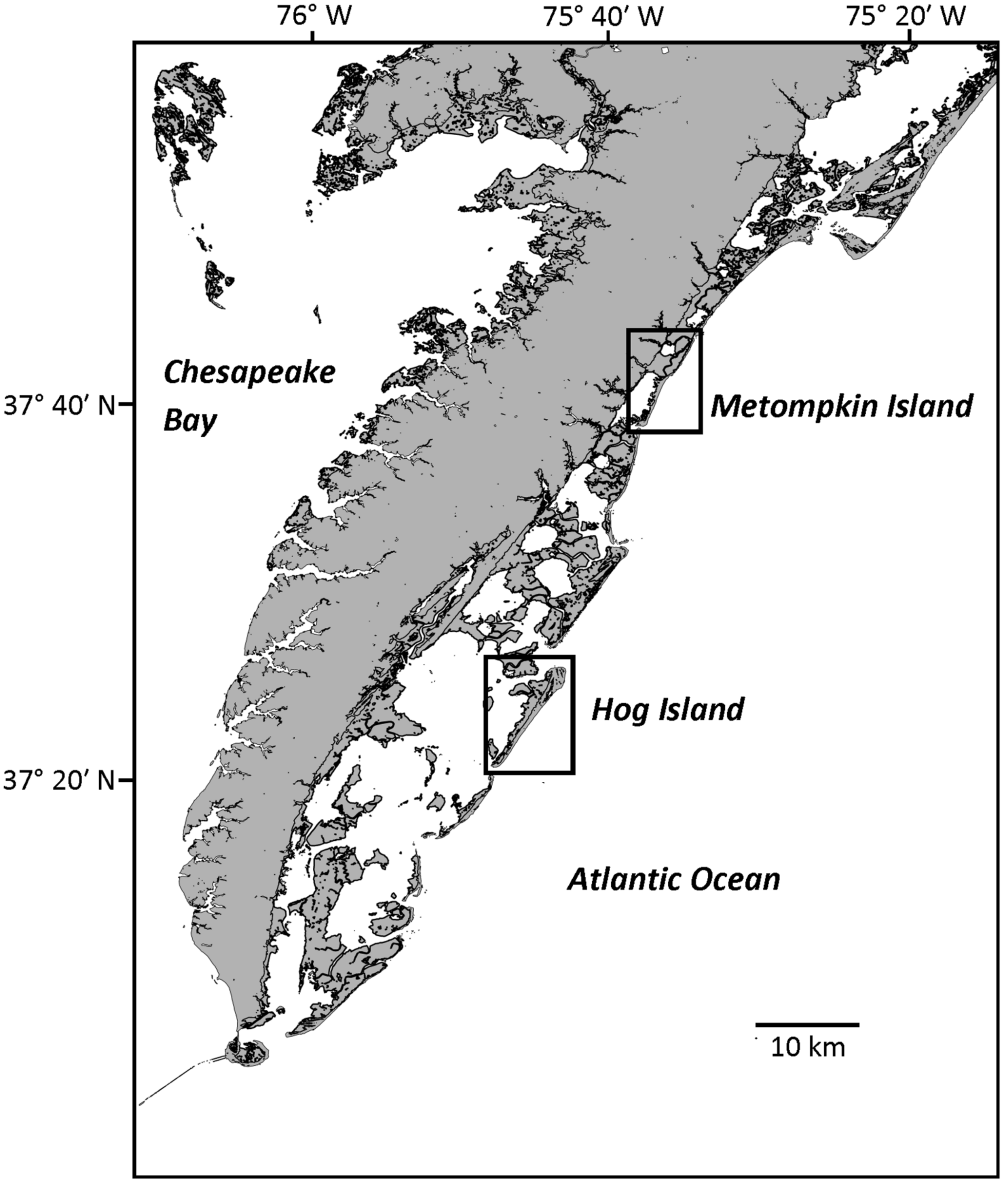
The Eastern Shore of Virginia, part of the DelMarVa peninsula on the Atlantic Coast of the Virginia, USA. Hog and Metompkin Islands represent end-members within the barrier island chain in regards to geomorphology, disturbance and vegetation. Hog Island has greater topographic complexity, is less frequently disturbed and has more plant cover and diversity than Metompkin Island.

Our study area was located on the Eastern Shore of Virginia, which represents the southern end of the Delmarva Peninsula between the Chesapeake Bay and Atlantic Ocean on the east coast of the United States. The Virginia Coast Reserve (VCR), a National Science Foundation funded Long-Term Ecological Research site (owned and managed by The Nature Conservancy) includes a chain of barrier islands that vary in size, shape, disturbance regime and vegetation coverage. Access to field sites was made possible by The Nature Conservancy (Lat. 37.417 N, Lon. 75.686 W) and U.S. Fish and Wildlife Services (Lat. 37.737 N, Lon. 75.563 W). No endangered or protected species were involved in the study. We focused on two islands that serve as end members in terms of island geomorphology (i.e. size, elevation, topographic complexity and disturbance history) within the Virginia barrier island system (Fig. 1, Table 1). Hog Island (Lat. 37.417 N, Lon. 75.686 W) is the larger, higher, and less frequently disturbed of the two islands. It is characterized by a series of shore-parallel dune ridges dominated by a mix of annual grasses and separated by dense shrub thickets [27], [28]. Mesic sites in the interior of the island are dominated by dense thickets of the evergreen, nitrogen-fixing shrub *Morella cerifera* L. Small (Myricaceae) while low elevation areas of the island interior are characterized by large areas of open water and freshwater marshes dominated by *Typha latifolia* L. (Typhaceae) and *Phragmites australis* (Cav.) Trin. Ex Steud. (Poaceae). Active overwash zones are generally limited to the first line of dunes with the exception of one large overwash zone near the center of the island, which may flood the island interior during strong tropical storms or intense Nor'easters. In comparison, Metompkin Island (Lat. 37.737 N, Lon. 75.563 W) is a smaller island dominated by relatively homogenous, low-lying topography at the northern end, whereas the southern end is characterized by discontinuous dunes. Vegetation is also homogenous and dominated by grasses with little woody diversity, most of which is located on the widest area of the island near the southern end. The simple topography and low elevation of this island make it prone to extensive overwash even during mild tropical storms and/or Nor'easters [29].

**Table 1:** Physical characteristics of two islands on the Atlantic Coast of Virginia, USA.

Island	Area (ha)	Length (km)	Width (km)		Mean Elevation (m)	Max Elevation (m)
			Min	Max		
Hog	750	12.1	0.3	1.8	2.3 (0.6)	5.8
Metompkin	295	10.4	0.2	0.7	1.7 (0.7)	4.7

Hog Island is the more topographically complex island with up to four dune ridges of varying heights and wider beaches whereas Metompkin Island has a single dune or no dune and narrow, gently sloping beaches. Mean elevation (± one standard deviation) includes data only from measurements collected during the current study and is limited to the primary dune and first swale. Max elevation for each island was derived from LiDAR imagery.

### Overwash identification

The study site had not been affected by a major hurricane since 2003 (Hurricane Isabel); however, several less intense storms impacted the region more recently including Tropical Storm Ernesto (2006), Tropical Storm Hannah (2008) and a particularly severe Nor'easter in Autumn 2009. The 2009 Nor'easter caused storm surges comparable to the record surges observed during Hurricane Isabel and had major impacts on dunes on both study islands. Specific overwash study sites were identified through ground surveys in spring 2010. Sites were classified as “active overwash” if they lacked relief and exhibited evidence of recent overwash such as lack of vegetation, wave-associated dune loss and/or hydraulic sand deposition within the past 1–2 years. Recently disturbed sites that showed some dune accretion since the most recent overwash event were classified “intermediate overwash”. Sites exhibiting evidence of overwash in the past several decades (e.g. shell armoring), but which were protected from wave action by seaward dunes at the time of the field survey were classified as “relict overwash”. In addition to sampling overwash sites, adjacent communities were sampled to determine species composition under undisturbed conditions. These sites were classified as either dune or swale based on elevation relative to the surrounding topography. Hereafter, these five environmental types will be referenced: active, intermediate, relict, dune, and swale. On Hog Island, six morphologically representative sites were identified: one large, active site; two intermediate sites; one relict site and two geomorphically stable sites with both dunes and swales. On Metompkin Island, six sites were identified: four active sites, one intermediate site, and one geomorphically stable site. Relict sites were not present on Metompkin Island. For each site, the physical extent of the overwash zone was estimated visually by walking the boundary of the overwash zone using indicators of overwash, such as hydraulic sand deposition (for recent overwash events), limited vegetation cover, presence of standing dead vegetation, consistently low topography and/or the presence of shells.

### Field sampling

During summer 2010, overwash community composition was sampled by visually estimating percent cover within quadrats along shore-perpendicular and alongshore transects. Many overwash zones were roughly semi-circular in shape (elongated in the cross-shore dimension) having a narrow neck extending landward from the beach toward the back-barrier. To best capture variations within this geometry, we established one shore-perpendicular transect (SPT) and two alongshore transects (AST) at each site. The SPTs extended from the toe of the foredune (or if dunes were absent from where the foredune toe was expected to be) to the first undisturbed community beyond the overwash zone. SPTs varied in length from 70 to 140 m. At each site, the ASTs intersected the SPT at 5 m and at the mid-point of the SPT, extending 50 m to each side of the SPT or until the first undisturbed habitat was reached. Together, the ASTs capture additional shore-perpendicular variation near the dune breach point and in the middle of the fan portion of the overwash. The adjacent undisturbed areas included grassy swales, intact dunes, mud flats, marshes and shrub thickets. All transects included one sampling point in adjacent undisturbed communities. Along each transect, vegetation was sampled every 5 m using a 0.25 m^2^ quadrat (0.5×0.5 m). Vegetation at each point was identified to the lowest possible taxonomic unit in the field according to [30], [31]. When positive field identification was uncertain, reference samples were collected and identified later according to [32]. Percent cover of each species was quantified visually to the nearest 5%, except for individuals of small plants which were recorded as 1% cover. No endangered or protected species were involved in the study. A list of species, with full scientific nomenclature can be found in Table 2.

**Table 2:** Species list for Hog and Metompkin Islands, two barrier islands on the Atlantic Coast of Virginia, USA.

Species (Authority)	Family	Habitat type								
		Metompkin Island				Hog Island				
		AO	IO	S	D	AO	IO	S	D	R
*Ammophila breviligulata* Fernald	Poaceae	X	X	X	X	X	X	X	X	X
*Borrichia frutescens* (L.) DC.	Asteraceae						X			
*Cakile edentula* (Bigelow) Hook	Brassicaceae	X	X	X	X	X	X		X	
*Cenchrus tribuloides* L.	Poaceae		X	X						
*Chamaesyce maculata* L. Small	Euphorbiaceae		X	X						X
*Cirsium* sp. Mill.	Asteraceae						X	X	X	X
*Conyza canadensis* (L.) Cronquist	Asteraceae			X			X	X	X	X
*Cyperus esculentis* L.	Cyperaceae		X	X	X		X	X	X	X
*Dichanthelium* sp.	Poaceae	X								
*Gnaphalium purpureum* L.	Asteraceae							X		
*Ipomoea* sp.	Convolvulaceae	X								
*Iva frutescens* L.	Asteraceae					X	X			
*Lepidium* L. sp.	Brassicaceae							X	X	X
*Morella cerifera* (L.) Small	Myricaceae						X	X	X	X
*Panicum amarum* Elliot	Poaceae	X	X	X	X	X	X	X	X	X
*Panicum dichotiflorum* Michx.	Poaceae		X	X			X	X	X	X
*Phragmites australis* (Cav.) Trin. ex Steud.	Poaceae	X	X	X	X	X	X			
*Physalis* sp. L.	Solanaceae							X	X	
*Rumex acetosella* L.	Polygonaceae						X	X	X	X
*Salicornia depressa* Standl.	Chenopodiaceae									
*Salsola kali* L.	Chenopodiaceae						X			
*Schizachyrium scoparium* (Michx.) Nash	Poaceae							X	X	X
*Scutellaria lateriflora* L.	Lamiaceae						X	X	X	
*Solidago sempervirens* L.	Asteraceae	X	X	X	X		X	X	X	X
*Spartina alterniflora* Loisel.	Poaceae	X								
*Spartina patens* (Aiton) Muhl.	Poaceae	X	X	X		X	X	X	X	X
*Strophostyles helvola* (L.) Elliot	Fabaceae	X					X			
*Suaeda maritima* (L.) Dumort.	Chenopodiaceae		X						X	
*Tragopogon dubius* Scop.	Asteraceae							X	X	

Abbreviations for habitat types are as follows: AO = active overwash zone, IO = intermediate or recovering overwash zone, D = intact dune, S = undisturbed swale, R = relict overwash zone.

The habitat type of each sampling point was classified as noted above. Location and elevation of each point were recorded with a high-resolution GPS (R7/8 GNSS, Trimble Navigation Limited, post-processed to cm-scale accuracy using OPUS). Location of the shoreline, defined here as the high water mark during a high tide, was also recorded for each site and distance to shoreline was determined for each sampling point. Sediment samples were collected every 10 m (every other sample point) on both the cross- and alongshore transects. After drying and sieving through a 2 mm sieve to remove shells, organic matter was removed using loss on ignition (LOI). Each sample was then analyzed in sets of 3 sub-samples using an LS 13 320 laser diffraction particle size analyzer (Beckman Coulter). The volume-percent size distributions of the sub-samples were analyzed to obtain the mean and standard deviation (which serves as a measure of sorting) for each site sampled.

### Data analysis

A total of 697 quadrats were sampled across both islands with 327 and 370 points on Hog and Metompkin islands, respectively. Species-sample curves were constructed to verify that the number of plots in each habitat type was adequate to represent species richness (i.e. the number of species present) for that habitat and island. Diversity was quantified for each habitat/island combination using the Shannon-Wiener Index applied to percent cover. To avoid pseudoreplication, spatial autocorrelation among sample points along each transect was tested by calculating Moran's *I* using distance and percent cover data. Although vegetation, especially coastal vegetation, often shows strong tendencies toward spatial autocorrelation, none was detected within these sites, likely due to the sparse cover, high habitat heterogeneity, relatively high species richness, and tendency for common species to occur among all plots. More importantly, this allowed each sample quadrat to be treated as an independent unit for the remainder of the analysis. Significant differences in percent cover among sites and among islands were determined by two-way ANOVA with Tukey post-hoc comparisons. Because relict overwash sites were absent on Metompkin Island, they were excluded from this analysis.

Canonical correspondence analysis (CCA) determined the potential influence of environmental characteristics on community structure [33]. The environment input matrix included elevation above mean sea-level, distance to the high water mark, beach width (defined as distance between dune toe and high water mark), mean grain size and the standard deviation of sand grain size at each sampling point. The community input matrix consisted of percent cover for each species in each quadrat. Row and column scores were standardized by centering and normalizing. Scaling was optimized by “site” (*sensu* PC-Ord) and scores were derived from “site attributes” for these ordinations. A Monte-Carlo test on Eigenvalues with 1000 iterations determined the significance of the relationship between matrices. Percentage of variation in community structure explained by each ordination axis was determined from a ratio of the axis Eigenvalue and the total variance [33]. The strength of relationship between each ordination axes and environmental variables are reported using the intraset correlations [33]. Separate analyses were performed for each island to examine island-specific associations between vegetation and environmental characteristics. A joint ordination was also performed to compare habitat classifications between islands. Scaling was optimized by species and axis scores were derived from species for the joint ordination. All ordinations were performed in PC-Ord Version 5 (MJM Software Design, Gleneden Beach, OR, USA). All data used herein are available on the VCR website at http://www.vcrlter.virginia.edu/home1/dataCatalog and can best be found using the dataset ID numbers VCR12186 through VCR12189.

## Results

We found a total of 29 species across all sites with the most common families being Poaceae (nine species) and Asteraceae (seven species) (Table 2). All but two species (*Morella cerifera* and *Iva frutescens*) were herbaceous (Table 2). Plant cover and diversity varied significantly along the disturbance chronosequence on both islands (Tables 3). On Hog Island, active overwash zones had the lowest cover and lowest diversity of all habitat types. All other habitats on Hog Island had ∼10 times more cover and at least twice the number of species as found in active sites (Table 3). On Metompkin Island, cover was also lowest in active sites but diversity in active sites was similar to diversity in intermediate and swale sites and higher than diversity on dunes. Dunes on Metompkin Island had the lowest species diversity of any habitat classification on either island (Table 3). In comparing the two islands, mean cover of all points was significantly higher (*p*<0.001, *F* = 7.059) on Hog Island than on Metompkin Island. On Hog Island, 23% of points sampled were bare sand and on Metompkin Island 46% of all sampled points were bare sand. Bare sand points were primarily located in active overwash zones on both islands. When bare points were excluded, mean cover was also significantly higher on Hog Island at 11.0±0.6% versus 9.0±0.5% on Metompkin Island (*p* = 0.010, *F* = 2.163) but the difference was smaller. Hog Island also had higher species richness with 25 species versus 16 species on Metompkin Island, and Hog Island had higher overall species diversity (Table 3).

**Table 3:** Summary of community characteristics for overwash zones and adjacent undisturbed dune and swale environments on two barrier islands along the Atlantic Coast of Virginia, USA.

	Hog Island			Metompkin Island		
Habitat	Species richness	Diversity (H′)	Cover (%)	Species richness	Diversity (H′)	Cover (%)
Active	6	0.60	1.0 (0.3) a, *	10	0.76	1.9 (0.3) a, *
Intermediate	17	1.01	12.4 (1.3) c, *	11	0.84	8.9 (1.4) b, c, *
Dune	17	0.84	9.7 (0.7) b, *	6	0.42	7.2 (0.9) b, *
Swale	16	1.01	9.7 (1.2) b	11	0.66	10.6 (0.9) c
Relict	13	0.86	10.2 (1.5)	–	–	–
All Sites	25	1.05	8.4 (0.5)*	16	0.90	4.2 (0.3)*

Species richness and diversity (Shannon Index, H′) values are calculated across all sampling quadrats within each habitat class. Cover is calculated as the mean (± standard error) for all sampling quadrats for a given class. An * denotes a significant difference in percent cover between similar habitats on the two islands. Lowercase letters denote significant differences among habitat types on the same island.

**Figure 2:**
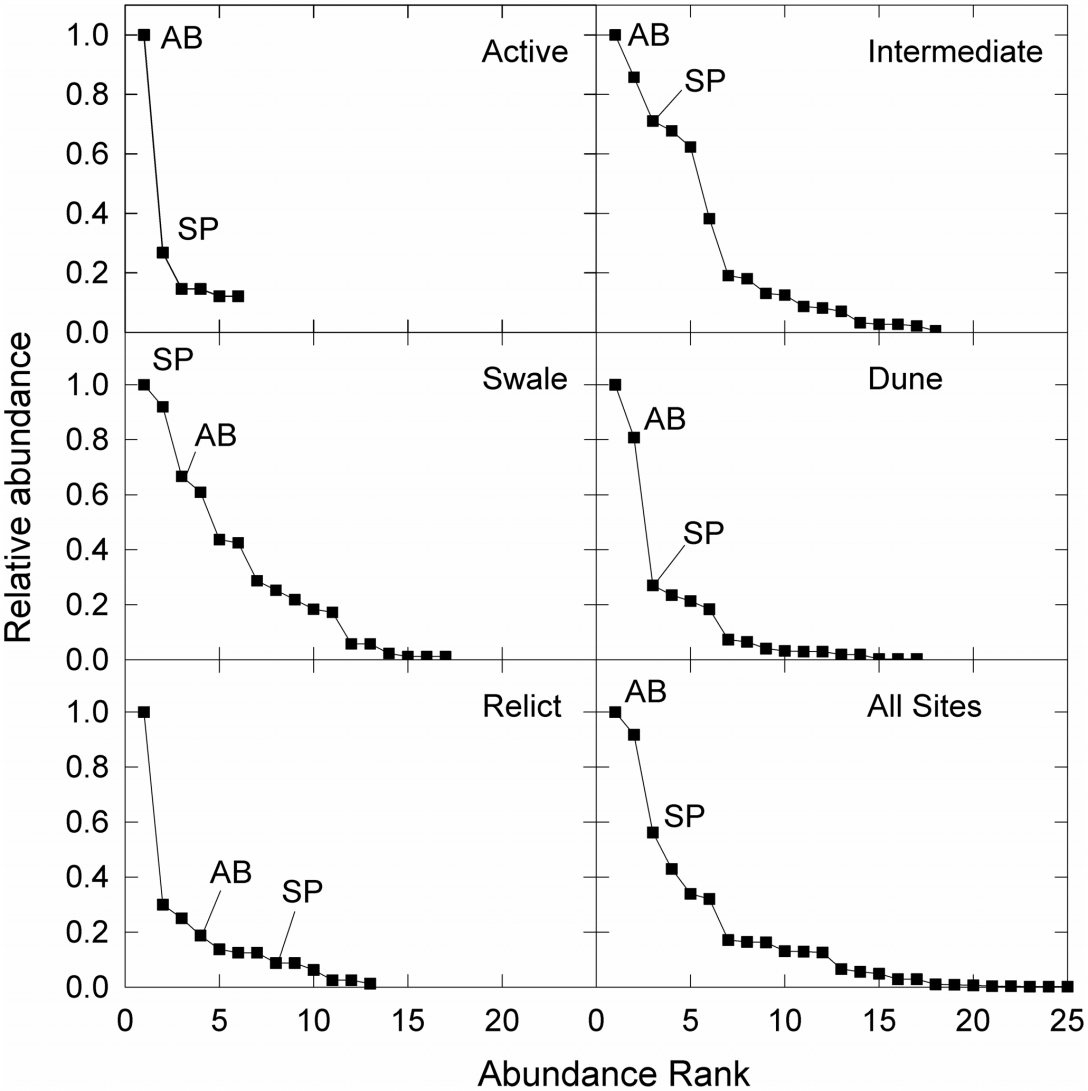
Rank abundance curves for five habitat types across a disturbance gradient and all samples on Hog Island, Virginia, USA. Ranks for *Ammophila breviligulata* (AB) and *Spartina patens* (SP) are noted on each panel. “Active” sites were recently overwashed while “intermediate” sites showed evidence of recent dune building after disturbance. “Relict” sites had historical evidence of overwash but are currently stable. “Dune” and “swale” sites are undisturbed habitats adjacent to overwash zones.

**Figure 3:**
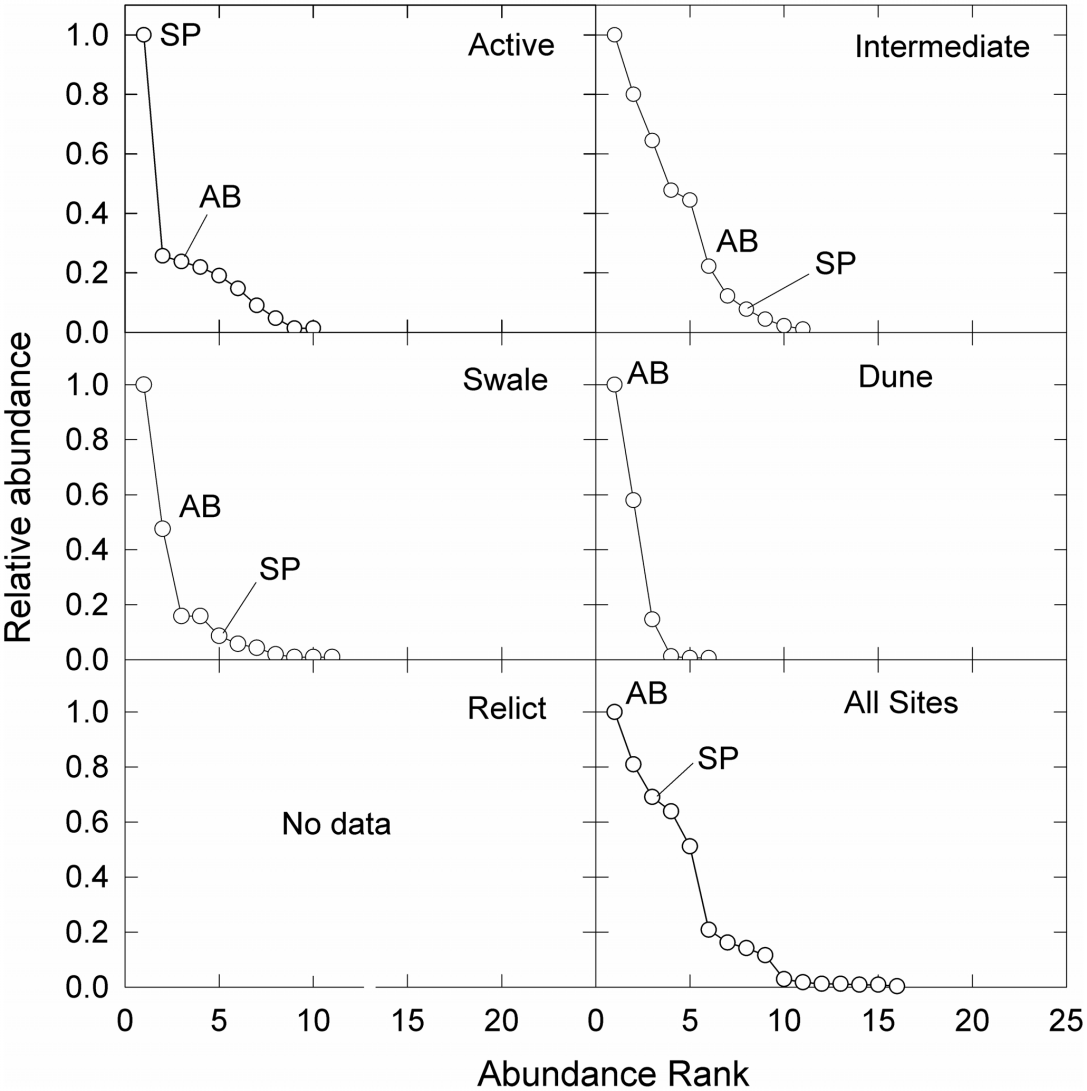
Rank abundance curves for four habitat types across a disturbance gradient and all samples on Metompkin Island, Virginia, USA. Ranks for *Ammophila breviligulata* (AB) and *Spartina patens* (SP) are noted on each panel. Note that Metompkin Island had no relict overwash sites. “Active” sites were recently overwashed while “intermediate” sites showed evidence of recent dune building after disturbance. “Relict” sites had historical evidence of overwash but are currently stable. “Dune” and “swale” sites are undisturbed habitats adjacent to overwash zones.

*Ammophila breviligulata* and *S. patens* were the most and third most abundant species, respectively, across all sites. *Panicum amarum* Elliott was also common on dunes and was the second most abundant species overall on both islands. When comparing cover of these species among habitat types, a typical pattern of *A. breviligulata* growth and dune recovery was observed on Hog Island—active overwash zones were dominated by bare sand, *A. breviligulata* was most abundant in active and intermediate overwash zones and on intact dunes, and *S. patens* was most abundant in low-elevation swales and relict overwash zones (Fig. 2 and Fig. 3). On Metompkin Island, active overwash zones were dominated either by bare sand or by sparse cover of *S. patens*. Although *S. patens* was the dominant species in active overwash zones, it was much less abundant overall on Metompkin Island and it was completely absent from dunes (Fig. 3). *Ammophila breviligulata* was the dominant species (i.e. highest frequency and abundance) on dunes (Fig. 3).

**Figure 4:**
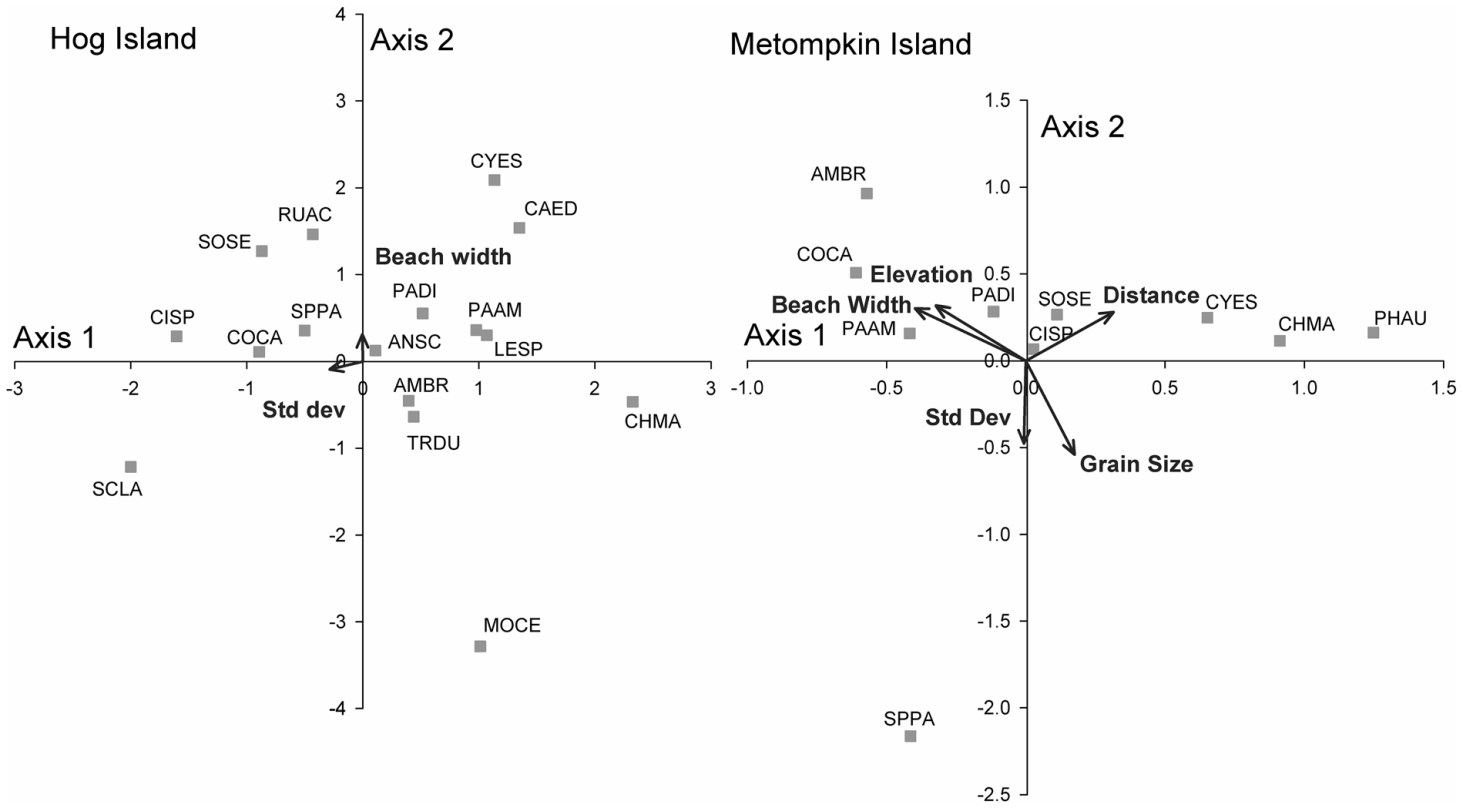
Canonical correspondence analysis of species scores and environment gradients on Hog Island (left panel) and Metompkin Island (right panel). Axes scores were derived from site attributes. “Bch_wdth” is the distance from the mean water line to the edge of the foredune, “Dist” is the distance from the sample plot to the high tide line, “Elev” is elevation above mean sea level, “Mean_Grn” is the mean sand particle size, and “Std_Dev” is a metric representing the heterogeneity of sand grain sizes at each point. Species codes: AMBR = *Ammophila breviligulata*, ANSC = *Andropogon scoparius*, CAED = *Cakile edentula*, CHMA = *Chamesyche maculata*, CIHO = *Cirsium horridulum*, COCA = *Conyza canadensis*, CYES = *Cyperus esculentes*, LESP = *Lepidium* sp., MOCE = Morella cerifera, PAAM = *Panicum amarum*, PADI = *Panicum dichotiflorum*, RUAC = *Rumex acetosella*, SPPA = *Spartina patens*, SOSE = *Solidago sempervirens*, SCLA = *Scutellaria lateriflora*, TRDU = *Tragopogon dubius*.

**Figure 5:**
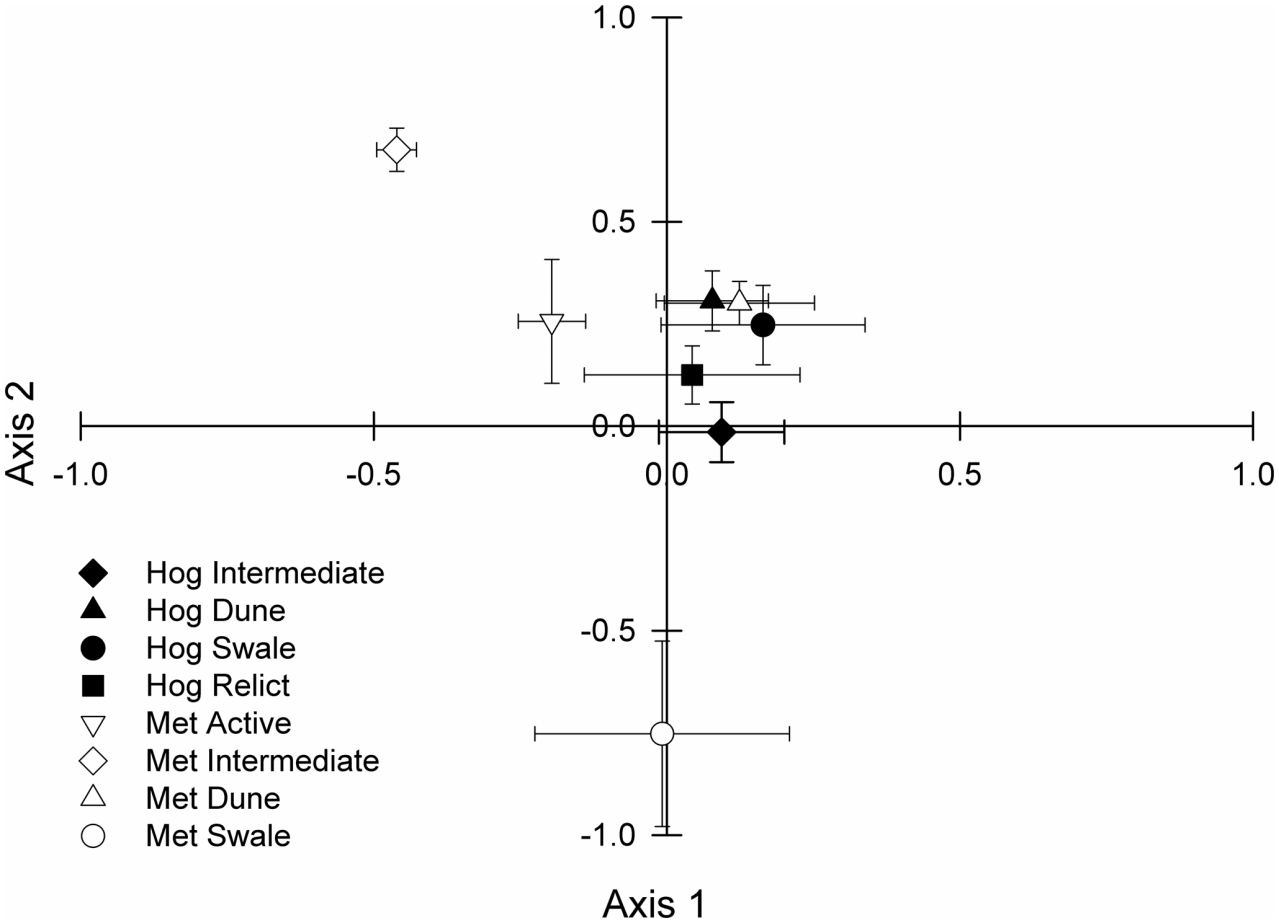
Mean (± SE) site scores from a joint ordination (canonical correspondence analysis) across five levels of overwash disturbance on two Virginia barrier islands, Hog and Metompkin (Met). Relict sites were absent from Metompkin Island. Axis scores were derived from species.

Environmental characteristics did not explain a significant amount of variation in community structure on Hog Island (*p* = 0.156 for Axis 1). Environment did explain a small, but significant amount of variation in community structure on Metompkin Island (Fig. 3) where Axis 1 explained 10.0% of the variation in community structure (*p* = 0.005) and Axis 2 explained an additional 6.5% of variation (*p* = 0.030). Axis 1 was negatively associated with beach width (*r* = −0.544) and elevation (*r* = −0.442) and positively associated with distance to shoreline (*r* = 0.432). Axis 2 was associated with sediment characteristics including mean grain size (*r* = −0.918) and grain size distribution (*r* = −0.809) (Fig. 4). When species ordination scores were plotted as mean values based on *a priori* habitat classifications (Fig. 5), all habitat classifications were relatively similar on Hog Island. However, habitat classifications on Metompkin Island diverged substantially, indicating greater variability in community structure on Metompkin Island than on Hog Island. Only dune sites exhibited any similarity between the two islands, likely because of the importance of *A. breviligulata* in dune sites on both islands (Fig. 5).

## Discussion

Differences in vegetation recovery among sites and between islands were not simply the result of variations in environmental characteristics that affect plant physiological tolerances. Rather, recovery of plant communities on barrier islands after storm overwash, and subsequent recovery of dunes, is a complex process that integrates site disturbance history (i.e. time since overwash and overwash frequency), island geomorphology, and the effects of local environmental characteristics. On Hog Island—the more geomorphically stable of the two islands—we observed a typical trajectory of vegetation establishment and dune recovery among overwash zones of varying ages [16], [17], [21] where *A. breviligulata* was the most abundant species in active overwash zones and intermediate overwash zones and the second most abundant species on dunes. Undisturbed swales were dominated by *S. patens* while relict overwash zones were dominated by a variety of later successional species that were unique to these sites. Metompkin Island—the more disturbed of the two islands— showed greater variability among habitats and a different pattern of plant community structure across the disturbance gradient. Dunes on Metompkin Island were dominated by *A. breviligulata*; however, the island was generally dominated by broad, low-elevation overwash terraces (often spanning the entire width of the island) that were dominated by *S. patens*.

The observed differences in species richness and species diversity between the islands and between similar habitats on each island suggest key differences in drivers of community structure. For instance, Hog Island had higher species richness than Metompkin Island overall; however, this was primarily due to the presence of an additional habitat type (relict overwash zones) that was not present on Metompkin Island. These sites were inhabited by several species not found in other habitat classifications and this explains much of the difference in overall richness between Metompkin Island and Hog Island. However, while overwash events temporarily removed most or all vegetation and few species can tolerate repeated overwash, species richness did not necessarily increase with persistent lack of disturbance. For example, five species (*Cenchrus tribuloides*, *Dichanthelium* sp., *Ipomoea* sp., *Salicornia depressa* and *Spartina alterniflora*) were unique to study sites on Metompkin Island, and all of these unique species were found only in active or intermediate overwash sites. Dispersal from overwash debris deposits (wrack) is an important source of propagules and promotes re-colonization of beach habitats after overwash [21], [34], [35], and supports theories that some level of disturbance promotes diversity [34], [35], [36], [37].

The observed patterns of species richness and diversity in undisturbed habitats also provide evidence for mechanisms of vegetation recovery after overwash. The least disturbed habitats on Hog Island (relict overwash) had the lowest species richness among habitats on Hog Island, further indicating that a lack of disturbance does not necessarily correlate with species richness, particularly if overwash deposits are a major source of propagules [34], [37]. More likely, species richness is primarily a function of dispersal [34], [36], [38] while cover and evenness are functions of stressors that filter out intolerant species [21] as well as time since disturbance. In less-disturbed habitats—such as relict overwash sites on Hog Island—a lack of new species introductions from overwash deposits and an increase in competitive filtering results in lower species richness, although diversity improves because no single species dominates, which is consistent with previous findings [34], [36], [37], [39].

Vegetation diversity, or a lack thereof, may also be an important indicator of the processes driving recovery of dune-building grasses. Dunes on Metompkin Island had the lowest diversity of any habitat classification on either island and were dominated by *A. breviligulata* (Table 3). *Spartina patens* was completely absent from dune sites on Metompkin. It is unlikely that *S. patens* was absent from dunes on Metompkin Island because of physiological tolerances. *Spartina patens* is common on dunes on Hog Island and may actually exclude *A. breviligulata* on older, less-disturbed dunes [28], [40]. Rather, the low diversity and absence of *S. patens* on dunes on Metompkin Island could indicate that *A. breviligulata* most easily establishes and grows to a sufficient density to build dunes when fewer competitors are present in the immediate area, especially the burial tolerant stabilizers [5]. *Ammophila breviligualata* did not seem to be negatively associated with other common dune species, especially *P. amarum*. Although the extremely low cover observed in our data indicates that resource (e.g. water and nutrients) competition is unlikely, interference may affect establishment. In a concurrent and complementary study focusing primarily on the geomorphic dynamics of these sites, it was suggested that *S. patens*, even in small amounts, stabilizes surface sediments in active overwash zones effectively “starving” *A. breviligulata* of sand needed to build dunes, particularly if shell armoring is also present and acting to suppress aeolian transport [10].

Although coastal ecosystems are typically considered to be driven by physical gradients that directly determine species composition based on plant physiological tolerances [21], [23], [24], our results show that island geomorphology affects the relationship between community structure and environmental characteristics such as elevation. Our ordination analysis suggested that there was no effect of environmental characteristics within the habitats we sampled on Hog Island; thus, it appears that all species present in these communities on Hog Island are adapted to the suite of environmental variables represented within the sites sampled. The results for Hog Island in no way suggest that abiotic stresses are unimportant on islands in general; rather, environmental variation in the relatively narrow strip of vegetation affected by overwash on Hog Island may not be great enough to select among the species sampled in this study. In comparison, a small, but significant effect of environmental characteristics was observed on the more frequently disturbed Metompkin Island where there was greater variation among habitat classifications. Elevation, distance to shoreline, beach width, sediment grain size and sediment size distribution all had small, though significant, associations with community structure. *Spartina patens*, in particular, was associated with grain size and sediment size distribution. This finding is consistent with [41] who showed that mean grain size was an important characteristic in driving community structure on Mediterranean dunes. *Spartina patens* was also found to be a significant indicator species of active overwash zones on Metompkin Island [10]. Coarse, poorly sorted sand typified Metompkin Island overall and overwash zones in particular [10], and the relationship between sediment size characteristics and *S. patens* provides some additional evidence of an association between *S. patens* and active overwash zones.

Our results compare favorably to other studies of community structure in coastal dune systems. Soil organic matter and mean grain size were found to be the best predictors of community structure in dune vegetation along the Mediterranean Coast where soil parameters explained ∼16% of variation [41]. Other recent work in coastal dunes [9], [42], [43] has shown stronger effects of environment on plant community parameters. However, direct comparisons among studies are difficult because of the variety of analytical approaches used (e.g. non-metric multidimensional scaling (NMS), Principal Components Analysis (PCA), path analysis, cluster analysis), differences in community characteristics included in the analysis (e.g. species richness, productivity) and the specific environmental parameters measured (e.g. storm frequency, soil organic matter). In general, these studies show that vegetative cover and diversity increase with lower storm frequency, reduced exposure to wind and wave energy, and increased soil organic matter. We believe our results are the first to directly show an association between a species (*S. patens*), overwash zones [4] and parameters linked to overwash disturbance (sediment grain size).

Several factors likely contribute to the relatively low amount of variation explained (compared to NMS, PCA, etc.) in our study and other studies using CCA [41]. For example, the relatively low cover observed in dune ecosystems and the small size overwash fans relative to other landscape units (e.g. salt marshes and dune ridges) inherently provide fewer samples and a weaker signal than environments having higher cover and encompassing a larger area. Additionally, because CCA integrates between a correspondence analysis ordination and a multiple regression of those scores with environmental data to find the best fit between environmental and community data, the amount of variation explained by CCA (relative to other ordination techniques) is generally expected to be lower [33]. Unlike other ordination techniques, such as NMS or PCA, CCA ignores community structure that is not associated with environmental variables. While this leads to lower variation explained relative to these other ordination techniques, CCA is better able to identify the most important environmental factors for community data where other, more open ordination techniques may not [33].

The dominance of *S. patens* in active overwash zones, the absence of *S. patens* from dunes on Metompkin Island and the association of *S. patens* with sediment size characteristics support previous work suggesting that biotic feedbacks have a significant effect on the success of dune-building grasses after storm overwash [5]. Specifically, it has been suggested that a feedback loop, subsequently termed the “maintainer feedback”, may contribute to the long-term maintenance of the low-elevation, frequently disturbed environment which characterizes Metompkin Island [5], [10]. The absence of dune-building due to the presence of *S. patens* enhances the probability of repeated disturbances [5]. *Spartina patens* would thus act as the “maintainer species” [10] because it contributes to the maintenance of the low-elevation overwash terraces that allow a cycle of repeated storm overwash.

Our results add to the growing body of work [4], [5], [10] that demonstrate the importance of feedbacks between vegetation and the environment in barrier island ecosystems. Shoreline changes resulting from increases in storm frequency and rising sea level are likely to increase in the coming decades, making barrier islands and associated ecosystem services more vulnerable [44]. Additionally, to reduce the vulnerability of barrier islands to global change, islands should be managed for ecosystem function rather than maintained as physical structures [45]. However, function is difficult, if not impossible, to generalize across systems as complex and dynamic as barrier islands. Feedbacks between vegetation and topography are an integral part of island function and our data show that feedbacks need to be understood across a variety of island morphologies, even for islands in the same chain. For much of the U. S. East Coast, the factors that affect the density of *A. breviligulata* after storms are an important component of island recovery, and understanding what drives this process is a critical step in learning to manage barrier islands for function.
